# Tracheal myoepithelioma resected by using rigid bronchoscopy: a case report and review of the literature

**DOI:** 10.1186/s13019-022-01880-0

**Published:** 2022-05-23

**Authors:** Parviz Mardani, Kamyar Ebrahimi, Reza Shahriarirad, Bita Geramizadeh, Hooman Kamran, Tahmoores Niknam, Mohammad Bagher Khosravi, Pooya Vatankhah

**Affiliations:** 1 Thoracic and Vascular Surgery Research Center, Shiraz University of Medical Sciences, Shiraz, Iran; 2 Department of Surgery, Shiraz University of Medical Sciences, Shiraz, Iran; 3 Student Research Committee, Shiraz University of Medical Sciences, Shiraz, Iran; 4 Pulmonary and Thoracic Ward, Abu-Ali Sina Hospital, Shiraz University of Medical Sciences, Shiraz, Iran; 5 Department of Anesthesiology, Abu-Ali Sina Hospital, Shiraz University of Medical Sciences, Shiraz, Iran; 6 Shiraz Transplant Research Center (STRC), Shiraz University of Medical Sciences, Shiraz, Iran; 7 Department of Pathology, Shiraz University of Medical Sciences, Shiraz, Iran

**Keywords:** Myoepithelioma, Tracheal tumor, Rigid bronchoscopy, Grasper forceps, Fiberoptic bronchoscopy, Argon plasma coagulation

## Abstract

**Background:**

Endotracheal tumors are rare in the respiratory system. Myoepitheliomas are benign tumors, which are rarely reported in the respiratory system. Herein, we report a rare case of endotracheal myoepithelioma, which was resected by rigid bronchoscopy.

**Case presentation:**

A 36-year-old man, presenting with chest pain, dyspnea, stridor, and hemoptysis, was referred to our center with radiological features of near-total tracheal obstruction due to mass. Fiberoptic bronchoscopy with argon plasma coagulation and rigid bronchoscopy with grasper forceps was utilized to resect the mass. Pathological evaluation of the mass demonstrated myoepithelioma. The patient was discharged in good condition. Now, after 6 months, the patient is symptom-free with no evidence of tumor recurrence or re-growth.

**Conclusions:**

Despite being extremely rare, myoepithelioma should be considered a possible differential diagnosis for endotracheal tumors. Fiberoptic and rigid bronchoscopy management is an effective method for the resection of endotracheal tumors.

## Background

Myoepitheliomas are benign neoplasms derived from myoepithelial cells accounting for 1–1.5% of salivary gland tumors [1]. Since the first reported case of benign myoepithelioma by Sheldon WH [2] in 1943, several studies have been conducted reporting myoepitheliomas in salivary glands and other sites, including breast, sweat glands, and even bone [3–5]. However, they have rarely been presented in the respiratory tract.

Tracheal tumors include less than 0.2% of all tumors in the respiratory tract [6], while extremely rare cases of endotracheal myoepitheliomas have been reported in the literature [7–10]. Despite their benign nature, surgical intervention is necessary in most cases due to complications.

Herein, we report a case of endotracheal myoepithelioma, resected with fiberoptic bronchoscopy with argon plasma coagulation and rigid bronchoscopy with grasper forceps. We also reviewed the literature with regard to the myoepitheliomas of the trachea and surgical interventions, including rigid bronchoscopy, to excise endotracheal tumors.

## Case presentation

### History and examinations

The patient is a 36-year-old man without any significant past medical history, who came to our center due to dyspnea, chest pain, stridor, and hemoptysis. His symptoms had started about 8 months before his admission with dyspnea and stridor after exercise. The initial symptoms deteriorated over time, till 10 days before admission; he developed chest pain and hemoptysis, which led to his admission to the local hospital. Computed tomography (CT) scan, lung spirometry, and bronchoscopy were performed, respectively, which revealed a large tracheal mass in the middle part of the trachea. No biopsy was taken at that time due to a shortage of necessary equipment in the local hospital. The patient was referred to our center for further investigation and treatment.

**Fig. 1:**
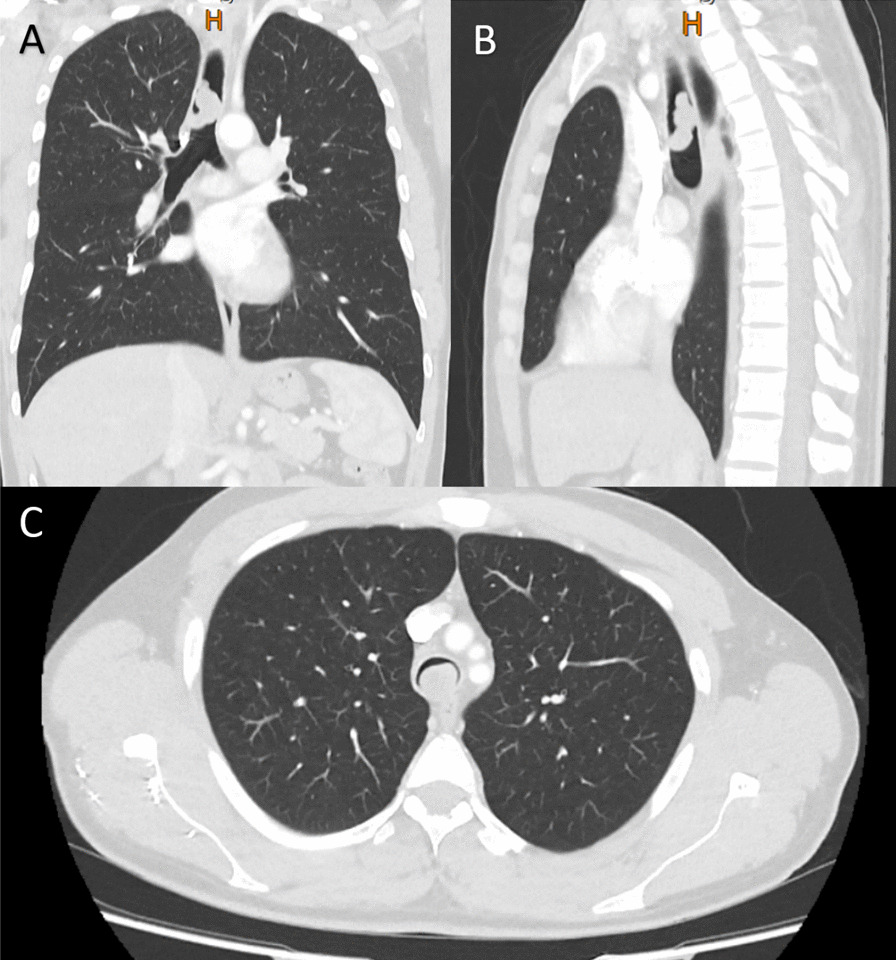
The computed tomography scan of a 38-year-old male with an endotracheal mass and near-totally obstructing the tracheal airway in its distal part; **A** coronal view, **B** sagittal view, and **C** axial view

**Fig. 2:**
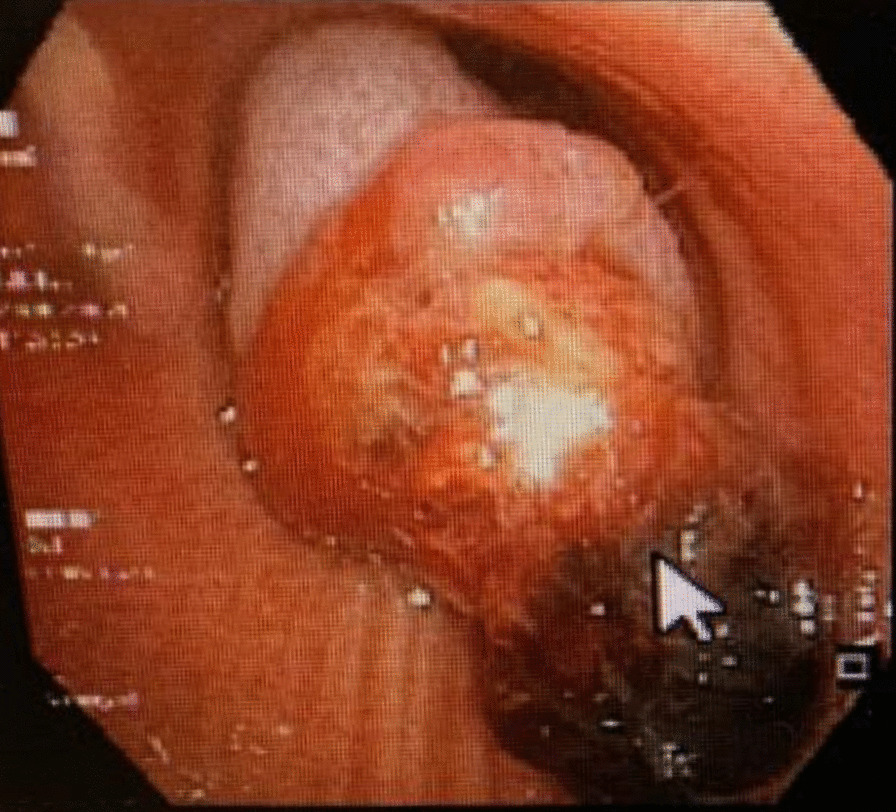
The bronchoscopy view of a large endotracheal mass (demonstrated with white pointer)

Upon his arrival, the patient was stable, and the only significant finding in his physical examination was mild stridor in lung auscultation. A high-resolution CT scan was done, which revealed a tracheal mass that nearly completely obstructed the airway (Fig. 1). Then, the patient underwent fiberoptic bronchoscopy for the evaluation of the mass, which confirmed the CT scan report, revealing an endotracheal mass in the distal part of the trachea causing near-total obstruction of the airway (Fig. 2). The patient was subsequently scheduled for surgical resection of the tumor.

### Surgical technique

**Fig. 3:**
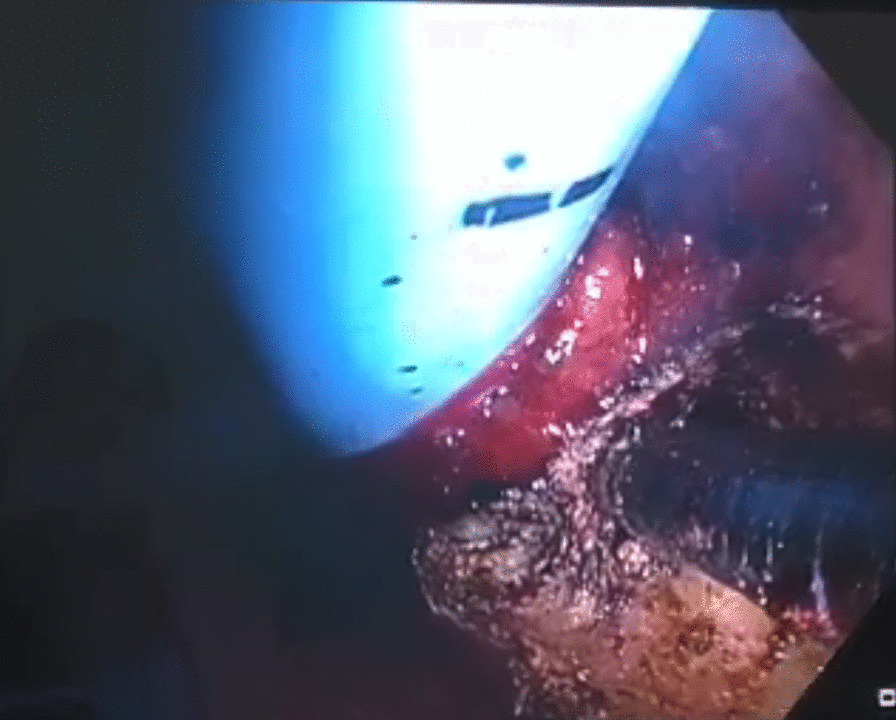
Utilization of argon plasma coagulation via fiberoptic bronchoscopy for partial resection of the endotracheal mass. The tracheal tube exchanger used for proper ventilation can be seen in this image

The patient was taken to the operating room for general anesthesia; then, an endotracheal tube exchanger, connected to jet ventilation, was inserted for proper ventilation. Surgical resection was planned with a bronchoscope. First, to control the bleeding, flexible fiberoptic bronchoscopy with argon plasma coagulation was utilized for partial resection of the tumor with laryngeal mask airway assist (Fig. 3).

Afterward, for complete resection of the remaining mass, since the endotracheal mass was exceptionally large and interfered with the patient’s breathing, rigid bronchoscope no. 8.5 was inserted to remove the mass. By utilizing grasper forceps and the bevel of the rigid bronchoscope, debulking and the resection of the tumor were attempted. After crushing the base of the mass, again, fiberoptic bronchoscopy with argon plasma coagulation was used via the rigid bronchoscope. In the same way, multiple attempts by using forceps graspers and fiberoptic bronchoscopy, and in the meanwhile, utilizing cautery and suction with the help of rigid bronchoscope, resulted in successful complete resection of the tumor with good hemostasis and bleeding control. Also, the sample of the mass was sent for pathology evaluation.

### Recovery and follow-up

**Fig. 4:**
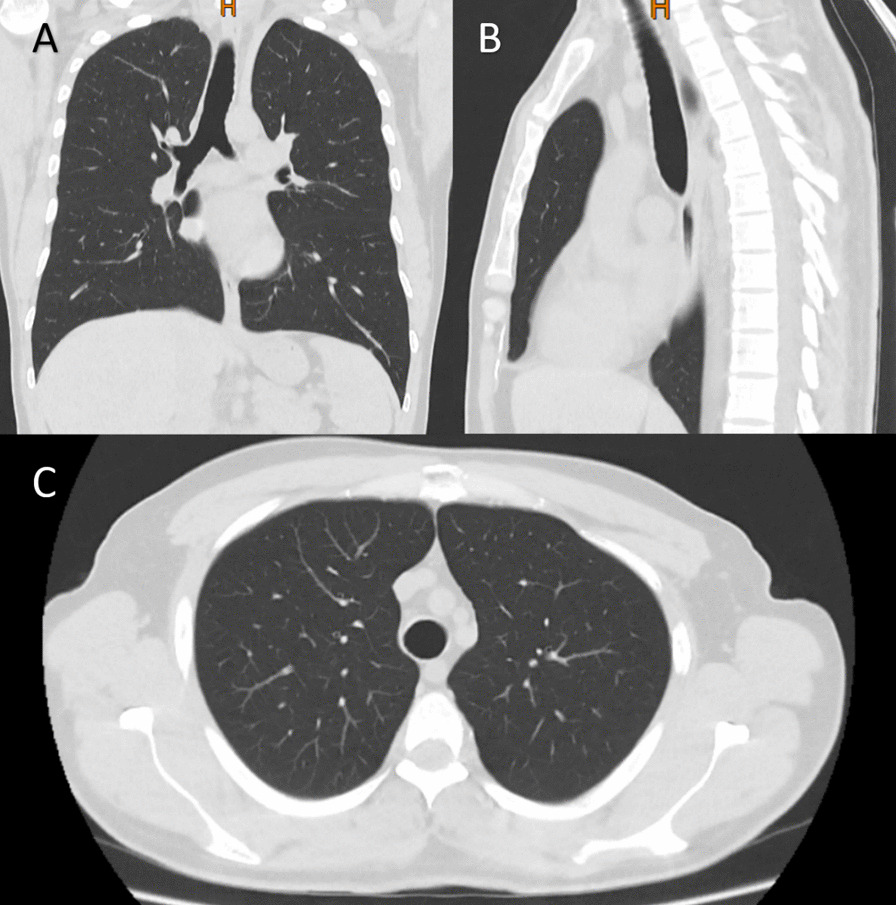
The computed tomography scan one month following surgical management of endotracheal tumor; **A** coronal view, **B** sagittal view, and **C** axial view

After the surgery, the patient was transferred to the intensive care unit for postoperative recovery, which was uneventful. In the follow-up, a CT scan and bronchoscopy were performed 1 month following the surgery, which showed no remnants of tumor remaining or regrowth with no bleeding or complications (Fig. 4). Also, he is currently under our follow-up.

### Pathology and immunohistochemistry evaluation

**Fig. 5:**
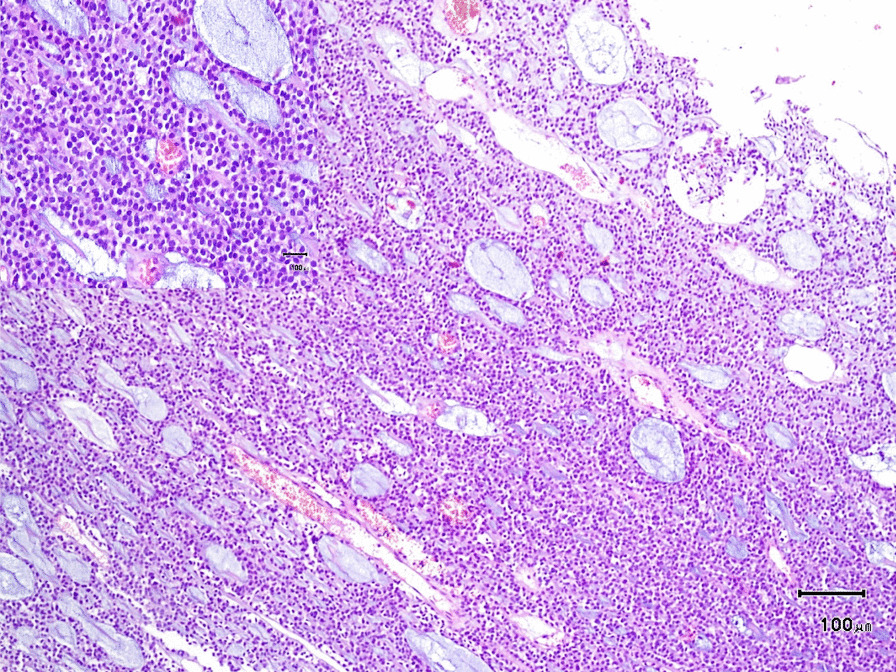
Pathological microscopic section, demonstrating solid and acinar plasmacytoid cells with myxoid stroma (H&EX250); inset shows high power view. No visible atypia or necrosis, and mitotic figures are low

The gross description of the sample showed multiple fragments of tissue with creamy gray color and soft consistency, measuring 2X2 cm. Histopathologic sections showed solid, myxoid and acinar patterns of myoepithelial cells with clear and plasmacytoid morphology, as well as a few ductal differentiations. Stroma showed mucoid and hyaline material. Also, no capsule was identified (Fig. 5). Furthermore, immunohistochemistry showed reactive cells for CK 7, SMA, and p63. The cells were negative for chromogranin, synaptophysin, CK 20, TTF1, napsin, S-100, EMA, CEA, and GFAP. The proliferation rate was low (Ki-67 was 8%). According to the above findings, the diagnosis of myoepithelioma was made.

## Discussion and conclusions

To the best of our knowledge, to date, only four cases of benign myoepitheliomas in the trachea have been reported in the literature, excluding our case [7–10]. Generally, primary tracheal tumors are rarely seen. In a study by Ahn et al. [11], they reported that malignant tracheal tumors include 0.5% of all thoracic malignancies, and benign tracheal tumors include only 1.16% of benign thoracic tumors during a period of 18 years in their center. Besides, regarding the main clinical manifestations of tracheal tumors, dyspnea and other airway obstruction symptoms may present [11]. Among these primary tracheal tumors, extremely few cases of myoepithelial tumors have been reported. Here, we reported a case of endotracheal myoepithelioma manifested with dyspnea, chest pain, stridor, and hemoptysis. After the resection of the tumor by fiberoptic and rigid bronchoscopy, clinical manifestations resolved, and no recurrence has been seen during 6 months following the surgery.

Regarding the resection of tracheal tumors, bronchoscopy can be a valuable tool [6, 12]. Here, we aimed to completely resect the tumor from the tracheal wall. Accordingly, we used a fiberoptic bronchoscopy approach. Argon plasma coagulation is a useful tool for the resection of benign tumors [13]; besides, rigid bronchoscopy helped us in exploiting our desired instruments, including cautery, flexible fiberoptic bronchoscope with argon plasma coagulation, and grasper forceps. This resulted in complete excision of the tumor with good hemostasis [6]. In addition, mechanical debulking was done via the bevel of the rigid bronchoscope. Regarding mechanical debulking, a study by Vishwanath et al. [12], reported 23 cases of tracheobronchial tumors who underwent rigid bronchoscopy. They concluded that rigid bronchoscopy and mechanical bulking is an effective therapy for airway obstruction. Also, in our case, for controlled ventilation during the surgery, a tracheal tube exchanger was connected to jet ventilation, and a rigid bronchoscope was passed alongside the tracheal tube exchanger [14].

Although myoepithelioma is defined as the benign neoplasm of myoepithelial cells, and on the other hand, myoepithelial carcinoma and malignant myoepithelioma are defined as malignant [4], *myoepithelioma* has been used for both benign and malignant neoplasms in the literature [15]. Therefore, the term *benign myoepithelioma* has been suggested to differentiate benign neoplasms from malignant ones [16].

Since Strickler et al. [17] presented the first case of myoepithelioma in the lung, only a few studies have been reported these neoplasms in the respiratory tract [16, 18, 19], with the lung, bronchus, and trachea as reported locations of the tumors in the pulmonary organs. To the best of our knowledge, excluding our case, only four cases of endotracheal myoepithelioma were reported in the literature [7–10]. Table 1 reviews clinical manifestations, pathology, immunohistochemistry, treatment, and follow-up of these cases.

**Table 1:** Literature review of endotracheal myoepitheliomas

Author, year	Age, sex	Tumor size	Signs and symptoms	Pathology	Immunohistochemistry	Treatment	Follow-up
Kim et al. 1998 [10]	38 y/o, female	N/A	Right neck mass	Spindle, epithelioid	S-100, SMA	Resection and anastomosis of the trachea with partial thyroidectomy	Well 8 months following the surgery
Chand et al. 2011 [8]	77 y/o, male	7 mm	Productive cough with blood-streaked sputum	Plasmacytoid	SMA, S-100, cytokeratin, AE-1/AE-3, CK 5/6, vimentin, calponin, focal positivity for p63	Flexible and rigid bronchoscopy and snare with cautery	N/A
Sekine et al. 2014 [7]	67 y/o, female	20 × 18 × 12 mm	Dry cough, dyspnea, wheeze	Spindle	Pan-cytokeratin, alpha-SMA, p63, S-100, GFAP, CD10, Ki-67 (4%)	Flexible bronchoscopy and snare with cautery	No recurrence 1.5 years following the surgery
Pfeiffer et al. 2018 [9]	10 y/o, female	1.4 × 1.1 × 1.9 cm (MRI)	Shortness of breath, tachypnea, accessory muscle use, hypoxia	Spindle, epithelioid	EMA, S-100, SMA, DOG-1	Tracheal resection with end-to-end anastomosis	No recurrence 2 years following the surgery
Current case	36 y/o, male	2 × 2 × 1 cm	Dyspnea, chest pain, stridor, hemoptysis	Plasmacytoid, clear	CK 7, SMA, p63, Ki-67 (8%)	Flexible fiberoptic bronchoscopy with argon plasma coagulation and rigid bronchoscopy with grasper forceps	No recurrence 5 months following the surgery

*Y/O* years old, *N/A* not available, *MRI* magnetic resonance imaging

For benign myoepitheliomas, a risk–benefit evaluation, like other nodules, is needed based on the clinical presentations, age, change in the lesion size, etc., to determine the need for treatment [8]. Regarding treatment, surgical resection is the gold standard [20]. For instance, surgical intervention is necessitated in conditions as our case, in which the location of the tumor causes near-total obstruction of the trachea that may lead to serious complications. As shown in Table 1, all previously recognized benign myoepitheliomas in the trachea have undergone surgical resection, of which two were excised with endoscopic snare and cautery [7, 8], and two were treated with resection and anastomosis of the trachea [9, 10]. It is worth mentioning that, in the case reported by Pfeiffer et al. [9], multiple recurrences occurred following multiple endoscopic procedures. So, they ended up performing tracheal resection with end-to-end anastomosis. As a result, they have suggested considering en bloc resection of the tumor with end-to-end tracheal anastomosis when facing these conditions.

An important differential diagnosis for benign myoepithelioma is its malignant form (malignant myoepithelioma or myoepithelial carcinoma). In soft tissue, atypia has been reported to be the only predictor for the malignant behavior of myoepithelial tumors; besides, high mitotic rates and necrosis are commonly seen in myoepithelial carcinoma [21]. In addition, pleomorphic adenoma or mixed tumor should be considered; myoepithelioma can be differentiated from pleomorphic adenoma by epithelial differentiation [22].

In conclusion, myoepithelioma of the trachea is reported extremely rare in the literature; however, it should be considered a differential diagnosis for respiratory tract tumors. For complete resection of endotracheal masses, fiberoptic bronchoscopy with argon plasma coagulation and rigid bronchoscopy with grasper forceps is an effective procedure.

## Data Availability

All data regarding this study has been reported in the manuscript. Please contact the corresponding author if you are interested in any further information.

## Abbreviations

CT: Computed tomography
Y/O: Years old
N/A: Not available
MRI: Magnetic resonance imaging
